# Gene‐centric analysis using functional annotation identifies genetic associations with blood‐based biomarkers of Alzheimer's disease in older adults from India

**DOI:** 10.1002/alz70855_099411

**Published:** 2025-12-23

**Authors:** Jennifer A Smith, Hasan Abu‐Amara, Wei Zhao, Zheng Li, Yuk Yee Leung, Gerald D. Schellenberg, Li‐San Wang, Priya Moorjani, Eileen M. Crimmins, Bharat Thyagarajan, Masroor Anwar, Peifeng Hu, Sharmistha Dey, Aparajit Ballav Dey, Xiang Zhou, Kumarasamy Thangaraj, Jinkook Lee, Sharon L R Kardia

**Affiliations:** ^1^ University of Michigan School of Public Health, Ann Arbor, MI, USA; ^2^ University of Michigan Institute for Social Research, Ann Arbor, MI, USA; ^3^ Penn Neurodegeneration Genomics Center, Department of Pathology and Laboratory Medicine, Perelman School of Medicine, University of Pennsylvania, Philadelphia, PA, USA; ^4^ Penn Neurodegeneration Genomics Center, Department of Pathology and Laboratory Medicine, University of Pennsylvania Perelman School of Medicine, Philadelphia, PA, USA; ^5^ University of California, Berkeley, Berkeley, CA, USA; ^6^ University of Southern California, Los Angeles, CA, USA; ^7^ University of Minnesota, Minneapolis, MN, USA; ^8^ All India Institute of Medical Sciences, New Delhi, Delhi, India; ^9^ University of California Los Angeles, Los Angeles, CA, USA; ^10^ Centre for Cellular & Molecular Biology, Hyderabad, India; ^11^ Center for Economic and Social Research, University of Southern California, Los Angeles, CA, USA

## Abstract

**Background:**

Alzheimer's disease (AD) and related dementias are a growing health and economic burden in India, the most populous country in the world. Blood levels of amyloid beta (Aß), tau, and other proteins marking neuronal injury are biomarkers of AD risk. Understanding genetic associations with blood biomarkers of AD in South Asians may help lead to improved prevention and treatment in this understudied population.

**Methods:**

We performed gene‐based analyses on 1) missense/loss‐of‐function single‐nucleotide variants (SNVs) and 2) promoter/brain‐specific enhancer SNVs across 84 genes previously associated with AD and 22 genes previously associated with AD biomarkers in the Harmonized Diagnostic Assessment of Dementia for the Longitudinal Aging Study in India (LASI‐DAD; *N* = 2,545) with the variant‐Set Test for Association using Annotation infoRmation (STAAR) across 7 AD biomarkers measured in blood: Aß40, Aß42, Aß42/Aß40, neurofilament light chain (NfL), glial fibrillary acidic protein (GFAP), tau phosphorylated at threonine 181 (pTau), and total tau (tTau). We adjusted for age, sex, and 10 genetic ancestry principal components, with random intercepts for genetic relatedness and biomarker plate. Analyses incorporated weighted annotation scores (e.g., deleteriousness). Significant results (FDR q<0.1) were followed up with single variant analysis to determine the SNVs driving the associations.

**Results:**

We found that 5 genes previously associated with AD (*ICA1L, SCIMP, APOE, TSPOAP1, CLU*) and 1 gene previously associated with AD biomarkers (*APOE*) were associated with pTau, Aß42/Aß40, and tTau in missense/LoF analysis in LASI‐DAD (FDR q<0.1). The top SNVs of *ICA1L, SCIMP*, and *TSPOAP1* (rs201355940, 17:5223434:C:T, rs144482724) were rare in LASI‐DAD (MAFs<0.005) and had high CADD Phred scores (11.2‐36.0) indicative of deleteriousness. In promoter/enhancer analysis, 1 AD‐associated gene (*CCDC6*) and 3 AD biomarker‐associated genes (*CCK, APOC1, SLIT3*) were associated with Aß40, Aß42/Aß40, and/or tTau. The top SNV in *CCDC6* (rs1171833) had a CADD Phred score of 19.6 and was common in LASI‐DAD (MAF=0.27). Several associated variants appeared to have an ancestral origin unique to India.

**Conclusions:**

Rare and common variants in AD‐ and AD biomarker‐associated genes may impact blood levels of AD biomarkers in South Asians. Gene‐based analysis may aid in identifying rare deleterious variants not seen in other populations.

